# Disparities in Utilization of Medical Specialists for Colonoscopy

**DOI:** 10.1089/heq.2019.0052

**Published:** 2019-09-03

**Authors:** Michele J. Josey, Cassie L. Odahowski, Whitney E. Zahnd, Mario Schootman, Jan M. Eberth

**Affiliations:** ^1^ Department of Epidemiology & Biostatistics, Arnold School of Public Health, University of South Carolina, Columbia, South Carolina.; ^2^ Cancer Prevention and Control Program, University of South Carolina, Columbia, South Carolina.; ^3^ Rural and Minority Health Research Center, University of South Carolina, Columbia, South Carolina.; ^4^ Department of Clinical Analytics and Insights, Center for Clinical Excellence, SSM Health System, St. Louis, Missouri.

**Keywords:** medical specialty, colonoscopy, screening, physicians, disparities

## Abstract

**Purpose:** Colonoscopy is the preferred screening modality for colorectal cancer (CRC) prevention. The quality of the procedure varies although medical specialists such as gastroenterologists and colorectal surgeons tend to have better outcomes. We aimed to determine whether there are demographic and clinical differences between those who received a colonoscopy from a specialist versus those who received a colonoscopy from a nonspecialist.

**Methods:** Using the population-based South Carolina Outpatient Ambulatory Surgery Database, we looked retrospectively to obtain patient-level endoscopy records from 2010 to 2014. We used multilevel logistic regression to model whether patients saw a specialist for their colonoscopy. The primary variables were patient race and insurance type, and an interaction by rurality was tested.

**Results:** Of the 392,285 patients included in the analysis, 81% saw a specialist for their colonoscopy. County of residence explained 30% of the variability in the outcome. Non-Hispanic black (OR=0.65; confidence interval [95% CI]: 0.64–0.67) and Hispanic patients (OR=0.75; 95% CI: 0.67–0.84) were significantly less likely than non-Hispanic white patients to see a specialist. Compared with commercial/HMO insurance, all other types were less likely to see a specialist, and even more so for rural patients. The interaction of race by rurality was not significant.

**Conclusions:** Specialists play a key role in CRC screening and can affect later downstream outcomes. This study has shown that ethnic minorities and adults with public or other insurance, particularly in rural areas, are most likely not to see a specialist. These results are consistent with disparities in CRC incidence, mortality, and survival.

## Introduction

Colorectal cancer (CRC) is the third most common cancer and the third leading cause of cancer-related death in the United States.^1^ CRC can be both prevented and detected at an earlier more treatable stage with receipt of regular recommended screenings. The overall CRC incidence and mortality have declined by at least 30% over the past two decades, due in large part to increased screening.^2,3^ However, the racial disparity between African Americans and white Americans in CRC mortality, and prevalence of adenomas and polyps (which are the precursors to CRC) have persisted.^4–7^ In addition to race, physician-related factors (e.g., medical specialty) and insurance coverage have also been associated with CRC screening and disease outcomes.

Colonoscopy is the preferred cancer prevention modality and was estimated to have contributed to a 77% and 65% reduction in CRC incidence and mortality, respectively.^8^ Colonoscopies are performed by various types of physicians, with varying degrees of quality. Specialty physicians complete additional clinical training in a specific area of medicine beyond their residency. For example, relevant to CRC screening, diagnosis, and treatment, gastroenterologists (GEs) and colorectal surgeons (CRSs) receive fellowship training beyond internal medicine and general surgery residency training, respectively. A colonoscopy is considered an invasive procedure that if not carefully done can have serious adverse effects (e.g., rare bowel perforation) or poor detection of potentially high-risk lesions.^9,10^ GEs are more likely than other specialties to remove adenomas and polyps during a colonoscopy.^4,11^ Research has also shown that having a GE perform the screening colonoscopy is associated with a lower rate of CRC after a negative colonoscopy.^12,13^ Furthermore, CRC patients experience better postoperative outcomes and overall survival when surgery was performed by a CRS than when surgery was performed by a general surgeon.^14,15^ Therefore, considering physician type is important when engaging in research on CRC outcomes.

Although most existing literature shows that specialists have better patient outcomes, specialty services are not equally accessible across population subgroups and geographic regions. For example, Medicare and Medicaid typically pay a lower reimbursement rate than private insurers, motivating physicians to prefer accepting new patients with private insurance.^16,17^ Referral patterns within a health care system or from independent clinics may also dictate the type of physician recommended for screenings such as colonoscopy.^18,19^ Insurance providers, particularly private insurers, typically have lower out-of-pocket costs for in-network providers. This could potentially limit the physician choices of lower income and rural patients because of higher out-of-pocket costs. Therefore, even when specialty physicians are available locally, some patients may experience barriers to care depending on insurance type.^20,21^

In addition to physician characteristics and insurance, urban residence is also associated with better patient outcomes.^22^ National studies have shown greater access to GEs and CRSs in urban counties than in rural areas.^23^ Likewise, GEs are located predominately in urban counties in South Carolina, whereas all the CRSs in the state were located in urban counties.^24^ Studies have shown that rural racial minority patients are often disadvantaged in the quality of cancer care they receive.^25,26^

Racial and socioeconomic disparities in CRC incidence and mortality have been well documented over time. Although CRC outcomes are dependent on physician type,^12–15^ there is a gap in the literature about differences in specialty utilization across population subgroups and geographic regions, particularly for colonoscopy. The purpose of this study was to determine whether there were demographic and clinical differences between those who received a colonoscopy from a specialist versus those who received a colonoscopy from a nonspecialist. We hypothesized that racial/ethnic minorities and patients without private insurance would be more likely to receive colonoscopy from a nonspecialist, and differences in specialist utilization would be further exacerbated by rural residence.

## Methods

### Study population

Data were obtained retrospectively from the South Carolina Outpatient Ambulatory Surgery Database (ASD), a population-based administrative data source with patient-level endoscopy records. We identified colonoscopy procedures using ICD-9, CPT/HCPCS codes (Appendix Table A1) between 2010 and 2014. Each record in the ASD had a unique patient identifier; demographic information including age, sex, race, insurance type, county, and ZIP code; and specialty information on the attending physician. If a patient had more than one colonoscopy during the study period, one record was randomly selected.

The target population for this study were adult patients aged 50–74 years with no personal history of CRC, which aligns with the population recommended for CRC screening by the Multi-Society Task Force on CRC.^27^ Patients were excluded if the ASD record indicated a colonoscopy was urgent/emergency, as the selection of the physician is likely beyond the patient's control.

### Outcome

The outcome for this study was whether patients saw a specialist or nonspecialist for their colonoscopy. A specialist was classified as a GE or CRS, and a nonspecialist physician otherwise. If medical specialty was missing [*n*=78,799 (4%) records], we used the 2009 and 2013 South Carolina Medical Board Directory to impute the specialty corresponding to the South Carolina license number. In addition, we used the National Provider Index to further supplement missing license numbers and medical specialty information [*n*=485,554 (26%) records]. The first physician listed on the colonoscopy record was considered the attending physician and the corresponding medical specialty was used when classifying the outcome. If the specialty of the first physician was missing and unable to be imputed, the specialty of the second physician was used, followed by the third specialty if the first two were missing. If the record contained no physician specialty information, it was excluded from the analysis (*n*=10,972).

### Primary independent variables and covariates

The primary patient variables of interest were race/ethnicity, insurance, and rurality. Race/ethnicity was categorized as non-Hispanic white, non-Hispanic black, Asian, Hispanic, or Other race. However, because the “Other” category was large [*n*=63,255 (16%)] and included no subclassifications in the ASD, the results from the “Other” category are not reported. Insurance was categorized as commercial/Health Maintenance Organization (HMO), Medicare, Medicaid, charitable, or Other (i.e., worker's compensation, other government insurance, or not stated). Rurality was categorized using the 2010 Rural-Urban Commuting Area (RUCA) Codes by the United States Department of Agriculture. RUCA codes categorize areas based upon their population density and commuting patterns.^28^ Patients were classified as either urban (1.0, 1.1, 2.0, 2.1, 3.0, 4.1, 5.1, 7.1, 8.1, 10.1) or rural (4.0, 5.0, 6.0, 7.0, 7.2, 8.0, 8.2, 9.0, 10.0, 10.2, 10.3).

The covariates considered for inclusion were chosen based on the literature and relationship with CRC: sex, family history of CRC, a personal history of colorectal polyps, having an inflammatory-related disease (Appendix Table A2), distance to the closest specialist, and ZIP code median household income. Distance was calculated as the straight-line distance from the patient ZIP code centroid (i.e., geographic center) to the nearest colonoscopy facility address. Median household income was obtained from the 2007 to 2011 American Community Survey, an ongoing survey performed by the U.S. Census Bureau that provides area-level sociodemographic information and categorized into tertiles.^29^ Patients were excluded if they had any missing covariates (*n*=10,972).

### Statistical analysis

We performed chi-square analysis and *t*-tests to compare demographic and clinical characteristics of patients who had a colonoscopy by a specialist versus patients who had a colonoscopy by a nonspecialist. We used multilevel multivariable logistic regression to model the odds of seeing a specialist. Because patients are nested within regions, we included patient county as a random effect. First, we ran an empty model that only included the random effect and calculated the intraclass correlation (ICC) to determine whether type of physician (specialty or not) varied across counties.^30^ Model 1 presents the relationship between physician type and each of the independent variables separately. Model 2 adjusts for the covariates and Model 3 includes an interaction term of rurality status between race and insurance. All models included the county-level random intercept. Interaction terms were tested to determine whether racial/ethnic or insurance differences were exacerbated by rurality. Data management and analyses were conducted in R version 3.5.2.

## Results

There were 392,285 patients included in the analysis and 81% saw a specialist for their colonoscopy. Although most patients saw a specialist, differences were present by race/ethnicity and insurance status. Table 1 shows that Asian patients had the highest proportion of colonoscopy performed by a specialist (85%), followed by white (81%), Hispanic (79%), and black (73%) patients. More commercial/HMO and charitable insurance holders (83%) saw a specialist than Medicare (80%), Medicaid (73%), and self-payers (73%). Urban patients were also more likely to see a specialist (82%) than rural patients (75%). Those having a history of a colorectal-related condition were more likely to see a specialist than patients with no history (*p*<0.001). There was also a positive association between ZIP code median household income and seeing a specialist (*p*<0.001); 89% of patients from the highest median income tertile saw a specialist versus 72% from the lowest median income tertile. Patients who had a colonoscopy in an ambulatory surgery center were significantly more likely to see a specialist than those who went to a hospital (91.0% vs. 68.4%, *p*<0.001).

**Table 1.** Characteristics of Study Participants That Received a Colonoscopy from 2010 to 2014, *n*=392,285

Characteristic	Total population, *N* (%)	Saw a specialist, *n* (%)	*p*
	392,285 (100)	318,369 (81.2)	
Race			
Asian	1655 (0.4)	1412 (85.3)	<0.001
Non-Hispanic black	73,942 (18.8)	53,877 (72.9)	
Non-Hispanic white	251,468 (64.1)	203,972 (81.1)	
Hispanic	1965 (0.5)	1556 (79.2)	
Other	63,255 (16.1)	57,552 (91.0)	
Insurance			
Commercial/HMO	216,716 (55.2)	178,701 (82.5)	<0.001
Medicare	133,140 (33.9)	106,128 (79.7)	
Medicaid	10,814 (2.8)	7842 (72.5)	
Self-pay	8144 (2.8)	5648 (73.0)	
Charitable	5835 (1.5)	1818 (82.6)	
Other	17,636 (4.5)	14,932 (84.7)	
Rurality			
Urban	365,649 (93.2)	298,466 (81.6)	<0.001
Rural	26,636 (6.8)	19,903 (74.7)	
Sex			
Male	174,102 (44.4)	139,521 (80.1)	<0.001
Female	218,183 (55.6)	178,848 (82.0)	
Age			
50–54	100,755 (25.7)	81,269 (80.7)	<0.001
55–59	79,680 (20.3)	64,114 (80.5)	
60–65	81,895 (20.9)	67,061 (81.9)	
65–69	77,389 (19.7)	62,861 (81.2)	
70–74	52,566 (13.4)	43,064 (81.9)	
Colorectal-related conditions			
Family history of CRC	49,414 (12.6)	41,116 (83.2)	<0.001
Colonic polyps	74,068 (18.9)	65,379 (88.3)	<0.001
Inflammatory disease	7382 (1.9)	6721 (91)	<0.001
Median ZIP code income ($)^a^	47,238 (13,197)	48,260 (13,181)	<0.001
≤$40,984	130,218	94,022 (72.2)	
$52,500–$40,985	137,282	113,752 (82.6)	
>$52,500	124,785	110,595 (88.6)	
Distance to closest specialist (miles)^a^	7.24 (5.24)	7.00 (5.09)	<0.001
Place of procedure			
Ambulatory surgery center	221,522 (56.5)	201,745 (91.1)	<0.001
Hospital	170,763 (43.5)	116,624 (68.3)	

Specialists were defined as gastroenterologist and colorectal surgeons. “Saw a specialist” column was calculated as the number of patients who saw a specialist divided by the total population of each demographic group. *p*-Values correspond to the “Saw a specialist” column.

^a^ Distance values are given as the mean (standard deviation) and was calculated as the straight-line distance from the patient ZIP code centroid to the closest physician specialist performing colonoscopy.

HMO, Health Maintenance Organization; CRC, colorectal cancer.

The empty model with the county-level random effect showed that 32% of the variability was explained by the patients' county of residence (ICC=0.321). Model 1 showed that non-Hispanic black and Hispanic patients had lower odds of seeing a specialist than non-Hispanic whites, and Asian patients had equivalent odds. Those with Medicare, Medicaid, or self-pay were significantly less likely to see a specialist than those with commercial/HMO insurance, whereas those with other insurance were more likely. Rural patients also had lower odds of seeing a specialist than their urban counterparts.

In the adjusted Model 2, non-Hispanic black (OR=0.65; confidence interval [95% CI]: 0.64–0.67) and Hispanic (OR=0.75; 95% CI: 0.67–0.84) patients remained significantly less likely to see a specialist than non-Hispanic white patients. Compared with patients with commercial/HMO insurance, patients who were self-paying (OR=0.61; 95% CI: 0.58–0.64), on Medicare (OR=0.87, 95% CI: 0.85–0.90), Medicaid (OR=0.74; 95% CI: 0.71–0.78), charitable assistance (OR=0.82; 95% CI: 0.76–0.88), or other insurance plans (OR=0.82; 95% CI: 0.78–0.86) were significantly less likely to see a specialist. The association between rural residence and seeing a specialist was slightly attenuated in the adjusted model (OR=0.93; 95% CI: (0.89–0.99). The odds of seeing a specialist increased with age, wherein patients 70 years or older had ∼11% higher odds than patients aged 50–55 years. Patients with a history of colorectal-related conditions were significantly more likely to see a specialist (Table 2).

**Table 2.** Odds Ratio (95% Confidence Interval) of Seeing a Specialist for South Carolina Residents for Colonoscopy, 2010–2014, *n*=392,285

	Model 1	Model 2
Race/ethnicity		
White	1.00	1.00
Black	0.60 (0.59–0.62)^*^	0.65 (0.64–0.67)^*^
Asian	1.01 (0.90–1.14)	1.06 (0.92–1.23)
Hispanic	0.67 (0.60–0.74)^*^	0.75 (0.67–0.84)^*^
Insurance		
Commercial/HMO	1.00	1.00
Self-pay	0.57 (0.54–0.61)^*^	0.61 (0.58–0.64)^*^
Medicare	0.91 (0.89–0.93)^*^	0.87 (0.85–0.90)^*^
Medicaid	0.63 (0.60–0.66)^*^	0.74 (0.71–0.78)^*^
Charitable	0.68 (0.63–0.73)^*^	0.82 (0.76–0.88)^*^
Other	0.88 (0.84–0.92)^*^	0.82 (0.78–0.86)^*^
Rurality		
Urban	1.00	1.00
Rural	0.91 (0.87–0.96)^**^	0.93 (0.89–0.99)^***^
Age		
50–54		1.00
55–59		0.99 (0.97–1.02)
60–64		1.06 (1.03–1.09)^*^
65–69		1.07 (1.04–1.10)^*^
70–75		1.11 (1.07–1.15)^*^
Sex		
Female		1.00
Male		0.82 (0.81–0.83)^*^
Colorectal-related conditions		
Family history of CRC		1.20 (1.17–1.23)^*^
History of colorectal polyps		1.82 (1.77–1.87)^*^
Inflammatory disease		2.76 (2.54–3.00)^*^
Median ZIP code income		
>$52,500		1.00
$52,500–$40,985		0.89 (0.87–0.92)^*^
≤$40,984		0.80 (0.77–0.82)^*^

^*^ *p*<0.0001, ^**^p<0.01, ^***^p<0.05.

The random effect was included as a random intercept for each county. Model 1 shows the relationship between physician type and each of the independent variables separately. Model 2 adjusts for the covariates. The intraclass correlation for the random effect in Models 2 and 3 was 0.296.

**FIG. 1.**
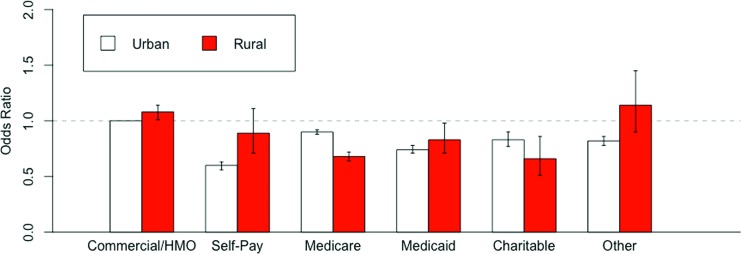
Adjusted odds ratio of seeing a specialist for the interaction of rurality with insurance.

The interaction of rurality with race/ethnicity was not statistically significant and not included in the final model, whereas the interaction with insurance revealed further differences. Rural patients who self-paid or had other insurance were not significantly different from urban patients who had commercial/HMO insurance. For the remaining insurance types, rural patients were similar or less likely to see a specialist than urban patients (Fig. 1). The ICC for the choice of the type of physician across counties was reduced only slightly when including all covariates (ICC=0.296).

## Discussion

We determined which sociodemographic and clinical characteristics were associated with utilizing a specialist for a colonoscopy procedure. Non-Hispanic black and Hispanic patients were less likely than non-Hispanic white patients to see a specialist, whereas Asian patients' utilization of specialists was no different than non-Hispanic white patients. Consistent with nationwide trends showing a declining availability of physician specialists in rural areas,^23,31^ rural patients were less likely to see a specialist for colonoscopy. Our hypothesis that rurality exacerbated disparities was confirmed for insurance status, but not for racial/ethnicity.

We found that non-Hispanic black patients had lower odds of seeing a specialist. In a study of male cancer survivors, Palmer et al. found that African American male cancer survivors were almost twice as likely not to have seen a specialist in the past 12 months than white survivors.^32^ Although black adults are slightly less likely to receive any CRC screening, an estimated one out of three men reported not receiving a screening recommendation from their physician.^33,34^ This lack of communication could transfer to disseminating the importance of physician selection for colonoscopy. Laiyemo et al. found no racial differences in pathological findings of diagnostic colonoscopies in the prostate, lung, colorecal, and ovarian trial, suggesting that observed disparities in CRC outcomes (e.g., lower adherence to follow-up testing and survival) may be due to access and health care utilization differences as opposed to biology.^35^ Similarly, a study on the Colorectal Cancer Prevention Network, a (free) screening program for low-income uninsured adults, found no racial differences in adenoma and polyp detection.^36^ All of the physicians who participated in the program were GEs. Our findings coupled with previous research suggest that racial disparities in CRC outcomes may be due in large part to access and utilization of high-quality screening and treatment. Interestingly, the odds that rural ethnic minorities saw a specialist were no different than their urban counterparts, even though specialty physicians are more prevalent in urban areas. Future studies should investigate whether this phenomenon is due to preference, lack of communication, or a byproduct of insurance coverage, as well as extend these findings to examine the effects of the receipt of colonoscopy from a specialist on long-term CRC outcomes.

Overall, patients who self-paid or had public insurance were less likely to receive a colonoscopy from a specialist. Apart from those with “Other” insurance, patients with insurance other than commercial/HMO were more likely to receive their colonoscopy in an outpatient hospital setting (data not shown). In South Carolina, 63% of GEs work primarily in ambulatory surgery centers and nonspecialists comprise a larger proportion of outpatient hospital-based endoscopy services.^24^ Our finding that Medicaid patients used a nonspecialist more than patients with commercial/HMO insurance may relate to the costs of performing the procedure. Unlike Medicare, coverage for Medicaid is not federally guaranteed for individuals not experiencing CRC-related symptoms.^37^

Finally, medical specialists who perform colonoscopy tend to perform a higher volume of procedures,^24,38^ which may be a major driving force behind better quality and colorectal outcomes.^39,40^ Some studies have found that despite volume or experience, GEs have superior quality outcomes.^11,41^ Other studies have shown that primary care physicians have the ability to have comparable metrics with colonoscopy specialists with the appropriate training and support.^42–44^ Although it may be difficult to disentangle the effect of medical training from procedure volume, accounting for the medical specialty of the physician or their procedure volume remains important in studies of CRC outcomes.

### Limitations and strengths

There are a few limitations to note. The data are an administrative-based resource, and our results are dependent on the accuracy of the data. Also, the available database did not have individual-level socioeconomic variables. A ZIP code-level variable was included instead, and it was shown to be a confounder and improved the model fit, a method that previous cancer studies have done.^45,46^ Despite these limitations, this study is innovative because we used a population-based data source (i.e., generalizable across the entire state) of adults who received a colonoscopy and had the power to detect subgroup differences across race/ethnicity, insurance status, and patient county of residence.

## Conclusion

This study illustrated how CRC disparities go beyond outcomes such as incidence and mortality, but also may exist in the provision of CRC testing. Specialists play a key role in CRC screening and can affect later downstream outcomes including improved survival.^12–15^ Our results point toward the need for targeted efforts to improve access and utilization of physician specialists for colonoscopy among racial/ethnic minorities and rural residents through expanding specialty practices into rural areas or improving rural access to urban specialists (e.g., mobile screening units and rotating practice sites). However, even in urban environments with greater availability of specialty physicians, racial/ethnic minorities, and those on public insurance may still be disadvantaged in accessing specialty care. Culturally tailored messaging for patients regarding how to access physician specialists (and why they are important) and self-advocacy in health care decisions may help to bridge the gap. In addition, system and policy changes to ensure equal access to physician specialists across insurance types may further mitigate observed differences.

## Abbreviations Used

ASD: Ambulatory Surgery Database
CI: confidence interval
CRC: colorectal cancer
CRSs: colorectal surgeons
GEs: gastroenterologists
ICC: intraclass correlation
RUCA: Rural-Urban Commuting Area

## Appendix

**Appendix Table A1.** CPT, HCPCS, and ICD-9 Codes Used to Identify Colonoscopy in Outpatient Data Set

Code	Description
G0105	Colorectal cancer screening; colonoscopy on individual at high risk
G0121	Colorectal cancer screening; colonoscopy on individual not meeting the criteria for high risk
44388	Colonoscopy through stoma
44389	Colonoscopy through stoma with biopsy
44390	Colonoscopy through stoma with foreign body removal
44391	Colonoscopy through stoma with control of bleeding
44392	Colonoscopy through stoma with hot biopsy
44393	Colonoscopy through stoma with ablation of tumor(s), polyp(s), or other lesion(s) not amenable to removal by hot biopsy forceps, bipolar cautery, or snare technique
44394	Colonoscopy through stoma with snare
44397	Colonoscopy through stoma with transendoscopic stent placement
45355	Transabdominal colonoscopy via colotomy
45378	Colonoscopy
45379	Colonoscopy with foreign body removal
45380	Colonoscopy with biopsy
45381	Colonoscopy with submucosal injection
45382	Colonoscopy with control of bleeding
45383	Colonoscopy with ablation of tumor(s), polyp(s), or other lesion(s) not amenable to removal by hot biopsy, forceps, bipolar cautery, or snare technique
45384	Colonoscopy with hot biopsy
45385	Colonoscopy with snare
45386	Colonoscopy with dilation
45387	Colonoscopy with transendoscopic stent placement
45391	Colonoscopy with endoscopic ultrasound
45392	Colonoscopy with endoscopic ultrasound with FNA
45.21	Transabdominal endoscopy of large intestine
45.22	Endoscopy of large intestine through artificial stoma
45.23	Colonoscopy
45.25	Endoscopic biopsy of large intestine
45.41	Excision of lesion or tissue of large intestine
45.42	Endoscopic polypectomy of large intestine
45.43	Endoscopic destruction of other lesion or tissue of large intestine
48.24	Endoscopic biopsy of rectum
48.36	Endoscopic polypectomy of rectum

**Appendix Table A2.** CPT, HCPCS, and ICD-9 Codes Used to Identify History of Colorectal-Related Diseases in Outpatient Data Set

Code	Description
Personal history	
V10.05	Personal history of malignant neoplasm of large intestine
V10.06	Personal history of malignant neoplasm of rectum, rectosigmoid junction, and anus
V12.72	Personal history of colonic polyps
Family history	
V16.0	Family history of malignant neoplasm of gastrointestinal tract
Inflammatory-related diseases	
555.0	Regional enteritis of small intestine
555.1	Regional enteritis of large intestine
555.2	Regional enteritis of small intestine with large intestine
555.9	Regional enteritis of unspecified site
556.0	Ulcerative (chronic) enterocolitis
556.1	Ulcerative (chronic) ileocolitis
556.2	Ulcerative (chronic) proctitis
556.3	Ulcerative (chronic) proctosigmoiditis
556.4	Pseudopolyposis of colon
556.5	Left-sided ulcerative (chronic) colitis
556.6	Universal ulcerative (chronic) colitis
556.8	Other ulcerative colitis
556.9	Ulcerative colitis, unspecified
564.1	Irritable bowel disease/syndrome
